# Clinical Implications of the Fascial System: A Commentary on One Surgeon’s Journey

**DOI:** 10.3390/life14010089

**Published:** 2024-01-05

**Authors:** Robert D. Rehnke

**Affiliations:** Private Practice of Plastic Surgery, Saint Petersburg, FL 33710, USA; robertrehnke@me.com

**Keywords:** fascia, self-organization, fractal, interstitium, basic fascial segment

## Abstract

A review of the most important concepts of the last 100 years on the topic of fascia and its fundamental importance to tissue and organ structure and function is provided as a basis for the author’s commentary on the self-organizing nature of fascia. Implications for clinical applications in medicine, in both pathophysiology and the treatment of disease, follow from these anatomic insights. Organizing principles of nature put forth by D’Arcy Thompson, Buckminster Fuller, Benoit Mandelbrot, and Adrian Bejan set the stage for understanding tissue and fascial form. The author presents videos from the operating room of living anatomy illustrating the concept of a basic fascia segment, which differentiates according to need in the various locations of the body.

## 1. Introduction

The twentieth century used a reductionist approach to teaching anatomy [1]. This concept of the body uses parts organized into systems: the musculoskeletal system, cardiovascular system, gastrointestinal system, etc. In the 20th century, cell biology focused on the cell with its nucleus, run using the genetic material contained within it, and ignored the extra-cellular matrix protein scaffold. The human body, however, is a complex system where the whole is greater than the sum of its parts. Fortunately, the 21st century teaches the human body as an integrated *whole*. A developmental paradigm that uses the “basic body segment” of the embryo and how it specialized into the adult form at every level is a good model of the wholeness of nature. Surgical training emphasizes dissection along fascial planes, which were relatively bloodless but overlooks how the connective tissue holds the body together. Examination of un-embalmed, fresh cadavers exposes the three-dimensional woven fabric described by Andreas Vesalius in the 16th century. In 1543, Vesalius published his opus magnum, “De Humani Corporis Fabrica Libri Septem”, which did not use the terminology of today, when referring to fascia, but instead called the superficial fascia the “fleshy membrane” [2,3]. Tumescent local anesthetic started as a technique for liposuction, but quickly, this injection of its dilute local anesthetic found its way into most surgical procedures performed in plastic surgery [4]. It not only lessens the narcotic requirement of surgery, but it also causes vasoconstriction that allows sharp dissection through tissue spaces enlarged by the injected volumes of fluid. Under these near-bloodless circumstances, it is much easier to see the fascia. The use of endoscopy in surgical procedures spread throughout surgical specialties at the end of the 20th century. The French plastic surgeon Jean-Claude Guimberteau used video as a research tool in his study of the fascia. 

His *Strolling Under the Skin* (https://www.youtube.com/watch?v=eW0lvOVKDxE accessed on 21 November 2023) is a revelation. 

Unlocking the mysteries of this fascial system is the secret to realizing the promise of regenerative medicine and cellular and biological therapies for the 21st century. 

What follows is a review of the contributions of some of the most influential minds in biology over the last century. D’Arcy Thompson taught in his book *On Growth and Form* that the laws of physics are in play in biology [5]. Stephan Levin, the American orthopedic surgeon, introduced the concept of *biotensegrity* to explain how the musculoskeletal system is pre-loaded with tensioned geometries governed by physics to support movement [6]. Graham Scarr collaborated with Dr. Levin to write an authoritative book on this topic [7]. Donald Ingber applied icosahedron tensegrity models to the cell’s cytoskeleton and explained its use for cellular-information processing, mechanochemical transduction, and morphogenetic regulation [8]. Stuart Kauffman was an early proponent of self-organizing complexity in biology. He wrote the book, “The Origins of Order”, and “At Home in the Universe: The Search for the Laws of Self-Organization and Complexity”, which explained the concept of self-organization, or how the spontaneous emergence of order is seen throughout nature [9,10]. This idea in biology that the whole is greater than the sum of its parts is known as *systems science* or *systems biology* and relates to a non-linear analysis of nature [11]. Another term in science which applies to biology and the nature of fascial organization has been called the edge of chaos or the complexity theory. It emphasizes interactions and the accompanying feedback loops that continuously change systems. Complex systems must have many interacting elements that yield uncertainty but do not have to be complicated. For instance, as a rule, the Tilapia fish will nest on the bottom of sandy lakes near one another (positive feedback loop). Still, they will not allow another fish to nest too close to theirs in crowded conditions (negative feedback loop) when there is a high enough concentration of fish in a lake. These two simple behavioral rules result in complex honeycombed patterns of nests on the bottom of lakes [12]. Complex systems may be unpredictable, but order-generating rules, like the laws of physics, constrain them. Most commonly in biology, feedback loops act through protein catalysts, which are the products of genes. 

Fascial tissue is the bedrock of the body. It shapes the organs and muscles, protects and nourishes the parenchymal cells, and is a repository of regenerative healing. This regeneration is self-organizing. This is not so hard to believe when one considers that the sperm and egg unite into a single cell, which will self-organize into a trillion-celled fetus. The twentieth century taught that adult tissue could not regenerate; twenty-first-century thinking challenges this concept. What is the basic structure and pattern of the self-organizing fascia responsible for the biological form and regeneration, and what are the implications for the clinician?

Most, if not all, forms in nature are laminated at the mesoscopic scale, whether you look at sedimentary-rock formation, stratus-cloud formation, or the stacked annual laminar layers of tree canopies, and animal tissue organization follows similar patterns.

Solid structures are formed in much the same way 3D printing works. A pattern is laid down in stacked layers of two-dimensional sheets held together with adhesive in a process known as *additive manufacturing* [13]. In this way, a Cartesian, three-dimensional architectural plan becomes a realized biological structure. Like architects and engineers plan high-rise buildings, macrophages and fibroblasts use biological building materials to create tissues, organs, and organisms in stacked layers or *floors*. A basic floor plan for each high-rise level is repeated until the top floor is completed. The complexity of self-organizing fascia must follow simple rules and patterns dictated by physical laws in that scale. As biology organizes itself, it must conform to efficiency laws to persist over time, that is to say, to stay alive. This is the explanation of biological evolution that Adrian Bejan uses to express the Constructal Law of design, which is one of the restricting rules of self-organization [14]. 

Carla Stecco wrote Functional Atlas of the Human Fascial System in 2015 [15]. Dr. Stecco, an orthopedic surgeon and professor of human anatomy and movement science at the University of Padua, Italy, based her findings on the dissections of hundreds of fresh human cadavers (they are magnificently illustrated in the photographs and histologic illustrations she recorded in her atlas). She stresses that fascia is a proper organ *system* with unique macroscopic and microscopic properties, functions, and pathologies. Dr. Stecco describes a pattern of superficial fascia midway between the dermis and deep fascia, with superficial adipose tissue (SAT) above and deep adipose tissue (DAT) below throughout the entire body. However, superficial fascia is a continuous system from the deep fascia to the dermis and is not limited to the few layers located halfway in this system.

The superficial fascia is organized into three main layers under the dermis and subcutaneous fat in most areas like the face and neck, chest and breast, and abdomen. Dr. Stecco gives evidence in her atlas that the deep fascia is organized into three layers of muscle groups in the chest, abdomen, back, and neck. She agrees with Thomas Myers, who stresses the complete organ-wide connectedness of fascia throughout the body. In his book “Anatomy Trains”, Meyers has an outstanding appendix on fascia [16]. 

Jean-Claude Guimberteau began using video endoscopy in 2005 to study the architecture of living tissue during more than 1000 surgical procedures. He benefited from video endoscopy’s ability to illuminate, magnify, and digitally record live surgical dissections. This gave him a unique perspective *between* the macroscopic and microscopic fields of anatomy and histology—the *mesoscopic* scale. He stressed his astonishment that cells do not occupy the entire body’s volume; he understood the importance of the extra-cellular matrix of proteins. Fascia shows that the extracellular *space* and its protein matrix, or ECM, are critically important body components [17]. 

## 2. Basic Fascia Segment

The basic-body-segment concept in biology states that complex bodies can be abstracted to simple basic plans, or segments [18]. In embryology the most basic segment is comprised of layers of ectoderm, mesoderm, and endoderm. Out of this most basic form, all the complex structures of ontology develop. Fascial tissues have a basic form as well, which is *laminated*. The basic fascial unit is composed of two-dimensional, diaphanous *sheets of fascia*, which are stacked one on top of the other and are held together with *vertical retinacula* that run at ninety degrees to the plane of the laminated sheets. But, here, we encounter an enigma. At the naked-eye level, surgical-dissection encounters separate sheets that, at the microscopic level, are seen at too small a scale to appreciate the overlying pattern; in truth, there are no *separate* sheets with *empty* spaces between them. The *mesoscopic* level, which exists between the naked eye and the microscopic level, is between 1× and 10× magnification. The mesoscopic-level organization of tissue and fascia, seen under high-resolution digital photography and computer enlargement, is highly important to our understanding of the body’s living tissue. This is where Guimberteau has taken us with his digital surgical video of in vivo tissue anatomy. Our inability to see these patterns with the naked eye or microscopic lens was waiting for the technology that is in everyone’s smartphone camera to arrive. It exposes the laminated nature of fascia and the continuum of fibers, collagenous sheets, and cells that are the amalgam of this most-important system. 

Histology is microscopic anatomy. However, fixation techniques required for histologic analysis are analogous to formaldehyde fixation in gross anatomic examination. Both techniques lead to distortions of fascia and have a blind spot to certain patterns that can only be seen in vivo. Later, we will encounter how important this is in appreciating the *interstitium*, as described by Dr. Neil Theise, a pathologist who uses in vivo microscopy with a technology known as Probe-based Confocal Laser Endomicroscopy (pCLE) to elucidate the true nature of the interstitium’s histology. 

**Figure 1:**
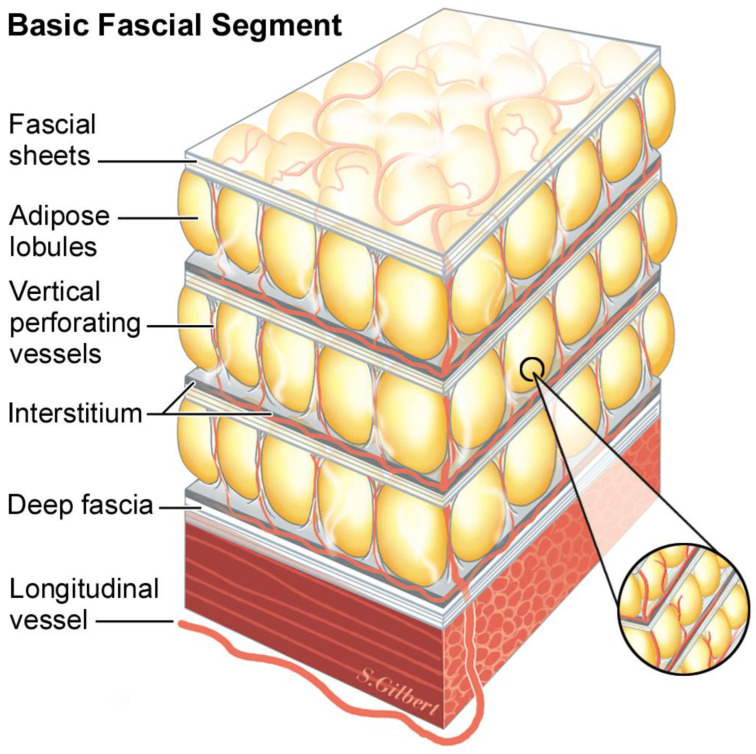
The basic fascia segment: stacked layers of 2D fascia held together with vertical retinaculum.

*Vertical retinacula* run ninety degrees to the plane of 2D stacked fascial sheets. But, just as the fascial sheets can be so thin and diaphanous that they are virtually invisible, the true three-dimensional structure of the retinacula is not usually seen. A common pattern in adipose layers is composed of lobules of adipose tissue held in the egg-crate-like structure of the vertical retinaculum. It is a three-dimensional cup that holds the adipose lobule, and when it abuts the other surrounding lobule/retinaculum units, the emergent honey-comb pattern makes the confluence of adjoining corners appear thick and as visible cords running to the dermis. Because each layer in the repeated architecture has a basal layer of interstitial fascia before encountering the next fascial sheet, dynamic sliding is possible between the layers (Figure 1). 

**Figure 2:**
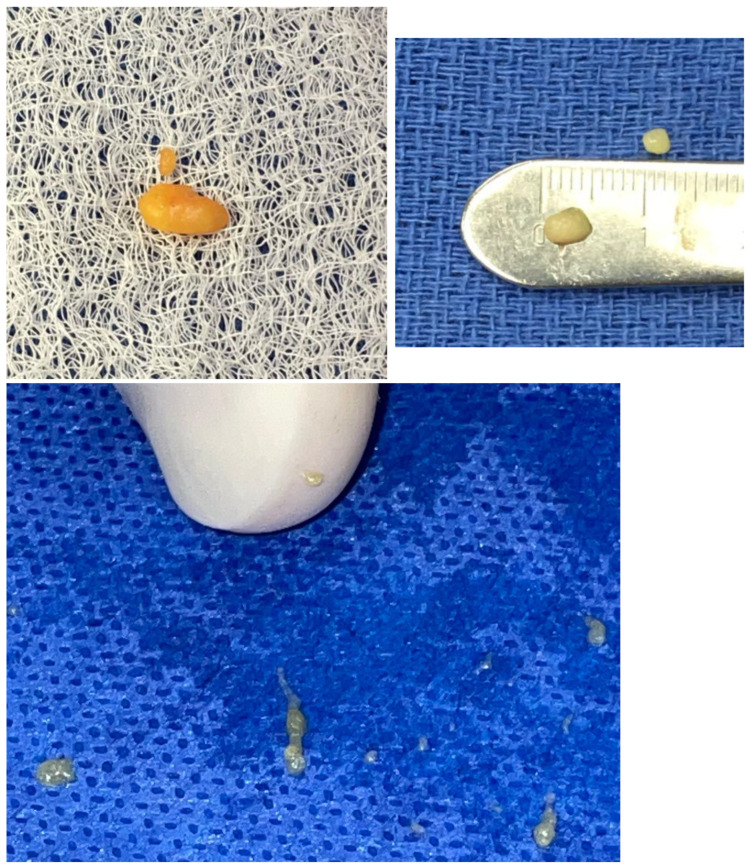
Fractal organization adipose lobules, compiled from close packing of micro lobules of various sizes and scales.

The basic model, repeated at different fractal scales, begins with a thin sheet of fascia, under which is a layer of differentiated tissue (adipose, muscle, epithelium, etc.), followed by a layer of interstitium. The organization of fat, from the single adipocyte to adipose tissue, is a fractal arrangement, or patterns of self-similar shapes, independent of the magnification scale. This mathematical concept applied to patterns in nature was first developed by Benoit Mandelbrot [19]. Fat cells are spherical and are close packed into small balls resembling icosahedrons. These balls are packed into micro lobules, with collagen skins that contain them. These micro lobules are packed together, forming varying-sized fat lobules depending on the body’s location and the patient’s body mass index (Figure 2).

**Figure 3:**
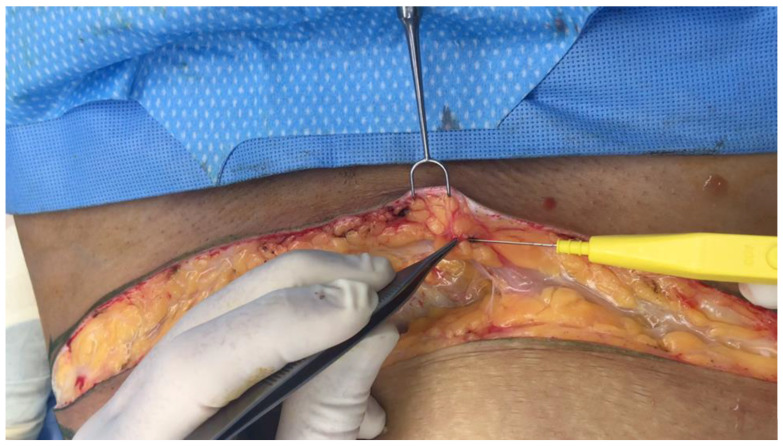
Supplemental Video S1, basic body segment—abdomen. The top of the video is caudal orientation, and the bottom is cephalic.

After the differentiated layer of cellular parenchyma, the final layer in the construction is the *interstitial* layer, then the fascia, differentiated tissue, and interstitium layers are repeated. Depending on the location in the body, more or fewer layers may be readily apparent due to the demands of that locale, which loads the fascia with stress, thickening and making the layer visible. These thickened layers, such as Camper’s and Scarpa’s fascia in the low abdomen, are frequently named; other layers are only seen easily with ultrasound and go unnoticed and unnamed (Figure 3). 

**Figure 4:**
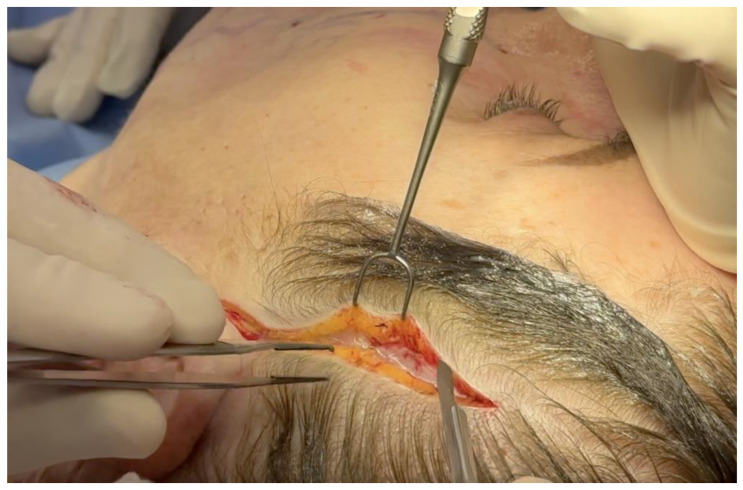
Supplemental Video S2, dissection through the multiple layers of superficial fascia in the temple after injection of tumescent local anesthetic. Note the injection of fat grafts into the spaces between the fascia is shown at the end of the video.

In other areas of the body, it is possible that differentiated tissue layers are unnecessary, and you find empty layers of fascia, such as those seen in the superficial temporal fascia (Figure 4). 

**Figure 5:**
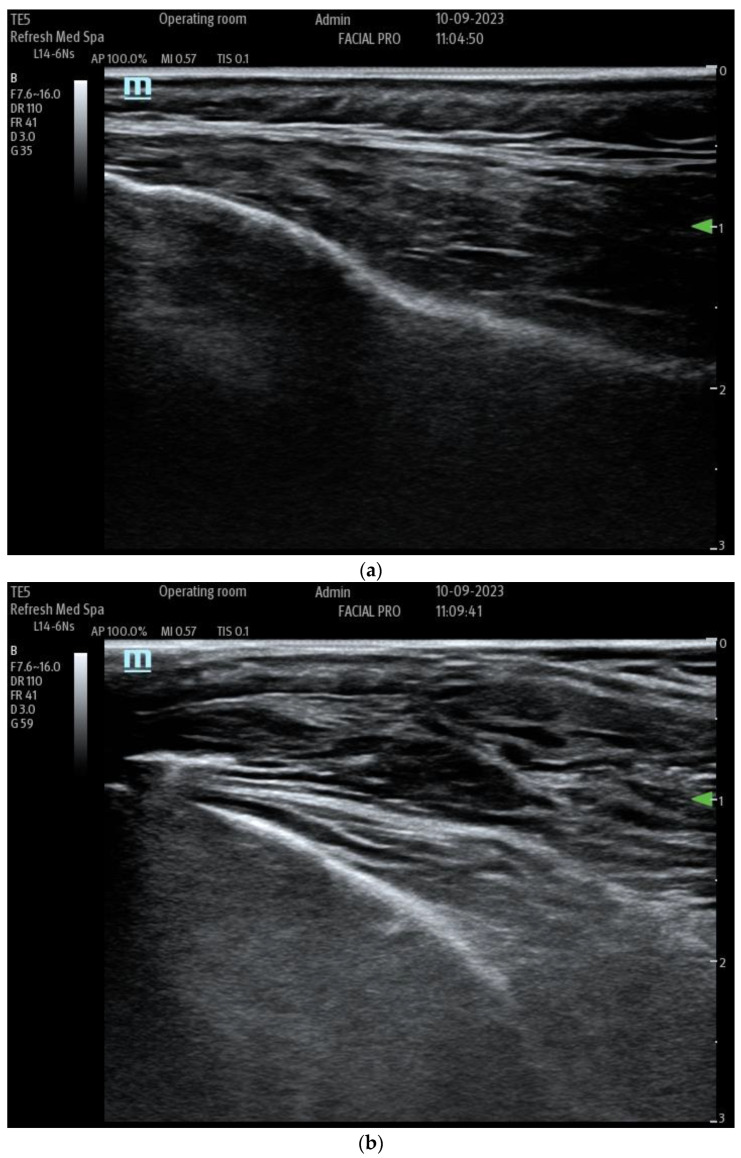
(**a**) Multiple layers of superficial fascia on top of the temporalis muscle. (**b**) Superficial Fascia of the temple after injection of dilute local anesthetic, which widens the separation between the layers of fascia.

Injecting tumescent anesthesia causes the layers to space farther apart, which can be seen in real-time with ultrasound (Figure 5a,b).

**Figure 6:**
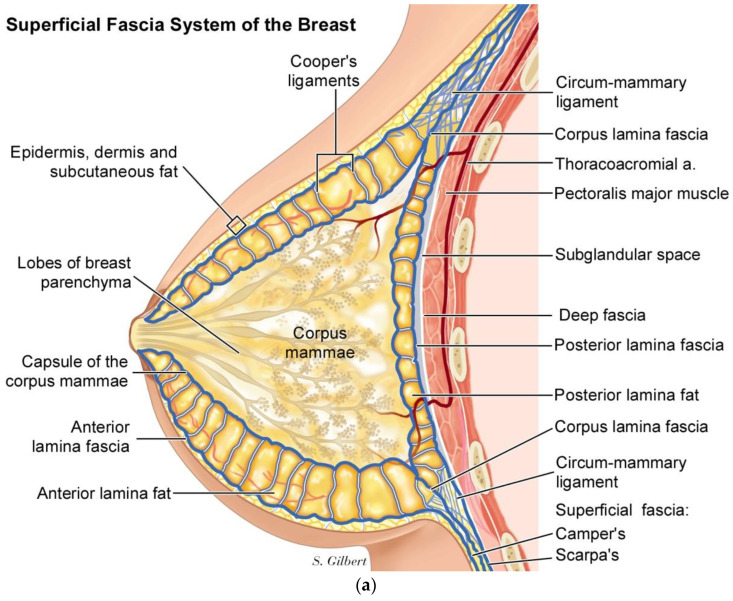

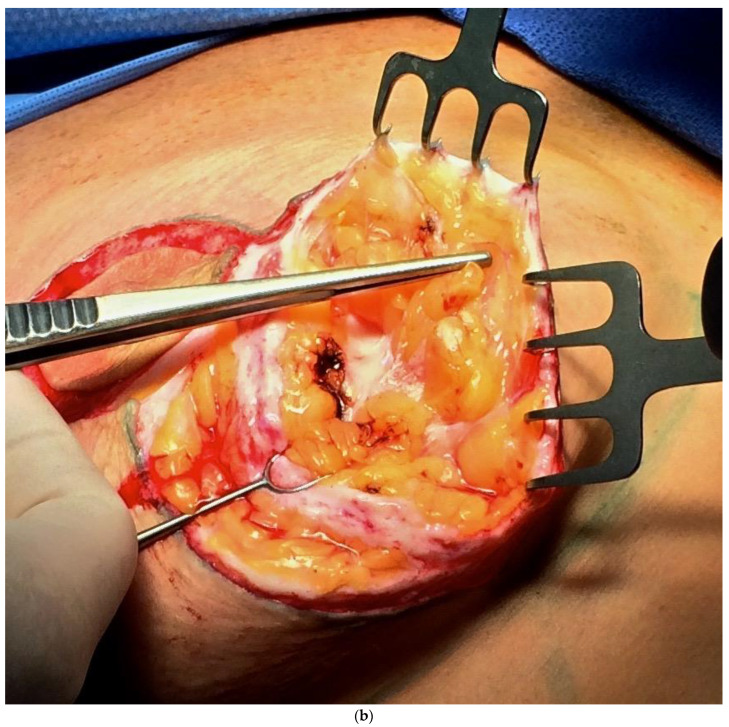
(**a**) Three layers of breast—Anterior, Corpus Mammae, Posterior Lamella. (**b**) Vertical mastopexy illustrating subcutaneous fat, anterior lamella fascia, and fat, white corpus mammae capsule, with Cooper’s ligament subdividing the fat lobules.

In the breast, we have published a superficial fascia system paradigm that includes- moving from superficial to deep, which is an anterior layer of fascia and fat, located just below the skin and subcutaneous fat, followed by the corpus mammae layer, which is contained within a collagen pseudo-capsule, and then a third layer of posterior lamella fat and fascia [20], (Figure 6a,b).

**Figure 7:**
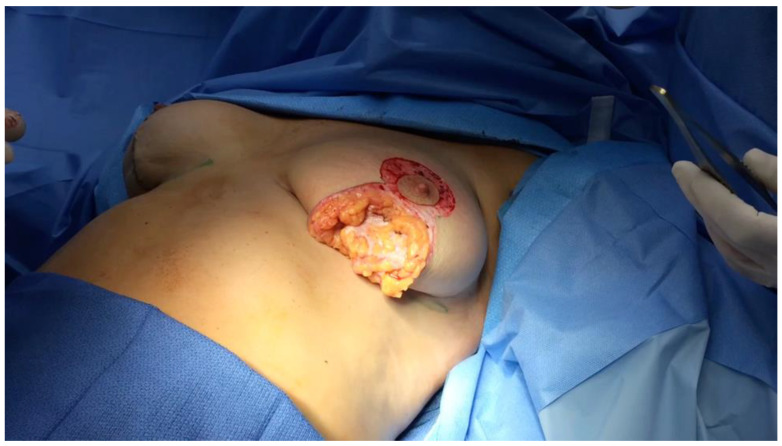
Supplemental Video S3, vertical mastopexy illustrating the superficial fascia system of the breast and circum-mammary ligament in a mastopexy using a cinching purse string suture to tighten the breast fascia.

The Corpus mammae is contained within a fusion of superficial and deep fascia surrounding the corpus mammae, called the circum-mammary ligament (Figure 7).

## 3. Interstitium

In 2018, Neil Theise’s group at NYU published a paper explaining the anatomic structure of interstitial tissue. Before this paradigm-changing gestalt shift, the interstitium was a theoretical necessity explaining the connection between capillary and lymphatic circulation. It is sometimes referred to as *loose areolar connective tissue*, which suggests that it is merely the simple “packing peanuts” of the body. For the first time, Theise and his colleagues, using novel technology (pCLE), could observe in vivo the structural relationships and characteristics of this sophisticated and ubiquitous *interstitial system*. They described “…previously unappreciated fluid-filled interstitial space, draining to lymph nodes, and supported by a complex network of thick collagen bundles. These bundles are intermittently lined on one side by fibroblast-like cells; there is a highly unusual and extensive unlined interface between the matrix proteins of the bundles and the surrounding fluid.” The interstitium is a pre-lymphatic space and is in continuity with the lymphatic system [21]. They described its presence in numerous tissues involved with intermittent or rhythmic compressive movement, including the submucosa of the entire gastrointestinal tract and urinary bladder, respiratory tract, dermis, peri-arterial soft tissues, and fascia. Theise identified interstitial lining cells, which do not meet the criteria for being endothelial cells or typical fibroblast. He described them as discontinuous lining cells, which may be precursors to myogenic fibroblast or stem cells. He speculated that they may be the cells that produce the proteins and glycoproteins found in the interstitium.

In their second publication on the interstitium, Theise’s group states, “Bodies have continuous reticular networks, comprising collagen, elastin, glycosaminoglycans, and other extracellular matrix proteins such as Hyaluronan, through all tissues and organs. Fibrous coverings of nerves and blood vessels create structural continuity beyond organ boundaries”. In this paper, they validated fluid flow through these fibrous tissues, showing evidence that supports their speculation that these spaces are in continuity body-wide [22]. 

Carla Stecco has described “fasciacytes”, which are hyaluronan-secreting cells she has found between the sublayers of aponeurotic fascia and the deep fascia, and the underlying muscle. Her description of the “loose areolar connective tissue” and the presence and function of hyaluronan and fasciacytes sounds exactly like Theise’s description regarding the interstitium [15]. It looks exactly like the dynamic structure of Guimberteau video tapes, which repeatedly show the dynamic formation of entangled polygonal-shaped, micellular branching tubes of glycoproteins in the interstitium [17,23]. They all underscore the importance of hyaluronan, which Theise uses as a marker of the interstitium. 

The interstitium is largely an *acellular* space if you do not count Theise’s interstitial lining cells or what Stecco calls fasciacytes. It is called “loose areolar connective tissue” in standard anatomic nomenclature. Theise and Stecco describe it similarly and teach that it is present throughout the body as a filler and supporter of cellular tissues. Guimberteau also points out its importance in the gliding movement of adjacent cellular tissues and the energy absorbing nature of its tensegrity system of proteins and glycoproteins. One can understand why it would have to be a part of the design for a *basic fascia segment*. This three-dimensional reticulated maze of acellular open spaces is filled primarily with collagen bundles, loosely arranged and filled with serum and glycoproteins such as Hyaluronan. Like Venetian canals, immune cells, and cancer cells traffic through the interstitium, a transportation zone. Its presence below the pulmonary and peri bronchial tissue, gastrointestinal, and genitourinary tract makes it a war front between the innate immune cells and invading infectious agents. It, however, is also important to the symbiotic functioning of the human microbiome (bacteria, fungi, protists, and viruses). 

Since hyaluronan is a marker for the interstitium, this provides an interesting clue about the interstitium’s role in embryogenesis and self-organizing regenerative healing after injury or surgery. 

Erich Blechschmidt, the renowned anatomist and embryologist, wrote his influential text, “The Ontogenetic Basis of Human Anatomy: A Biodynamic Approach to Development from Conception to Birth”, in 1978. It was translated into English in 2004 by Brian Freeman [24]. In the preface, Freeman states, “Chapter 1 establishes the book’s perspective and philosophical argument for choosing the biomechanical and biodynamical approach and introduces the ideas of growth movements, growth forces, and the metabolic field”. These concepts fit seamlessly with Ingber’s teachings on the tensegrity of the ECM and intracellular cytoskeleton. Brian Goodman, in his book “How the Leopard Changed Its Spots: The Evolution of Complexity”, describes a *Morphogenetic Field* as a precise field of physics at the cellular level. He and his collaborators studied how a cell controls calcium concentrations in the cytoplasm, and the influence it has on the mechanical properties of cytoskeletal proteins. They, along with others, believe these calcium–cytoskeleton interactions, “could result in the spontaneous formation of spatial patterns in the concentration of free calcium and the mechanical state of the cytoplasm, measured by strain (stretching or compression)”. They view this type of behavior as crucial for any model of morphogenesis [25]. 

Blechschmidt followed the tradition of Vesalius by looking for himself and studying *human* specimens, not animal embryos. His total reconstruction of embryos, prepared from specimens cut one millimeter in thickness, is called the Blechschmidt Collection of Human Embryos in the Anatomical Institute of Gottingen University. His collection contains embryos of different ages, which permit spatial comparisons between the developmental stages, thus permitting the demonstration of development movements. He puts forward the term *anlage*, the embryonic rudimentary foundation of a particular organ or anatomic part. Another one of his important concepts is the *biodynamic metabolic field,* which “…is a field of force based on a locally ordered metabolism. Metabolic fields are those morphologically definable regions at all different levels of spatial resolution, which contain spatially ordered metabolic movements. Biodynamic metabolic fields can be used to describe cells and cell ensembles (e.g., zones of loose tissue, zones of dense tissue) or whole areas of differentiation such as the lung, the liver, or the thyroid gland”. Blechschmidt states that embryonic morphogenesis requires both *spatial opportunity* and *metabolic occasion*. He makes a distinction between two basic types of tissue: *limiting tissue,* which is largely epithelium of ectodermal or endodermal origin, and *inner tissue*, which is exemplified by mesenchyme in the embryo, characterized by scattered cells in watery intercellular byproducts of metabolism contained in vacuoles of the interstices between the cells and collagen fibers. These inner tissue fibers can consolidate into intercellular substance fascicles in thin layers of fascial sheets or lattices of variable mesh sizes with highly deformable interstices. A particular inner-tissue field is called a *suction field.* Because they are located adjacent to the limiting field epithelium, they are also called a parathelial loosening field. Since the epithelium grows faster than the inner-tissue stroma, it separates and forms a cleft between the epithelium and its underlying mesenchymal stroma [24]. Blechschmidt stresses that these changes in the spatial arrangement of the cells making up a tissue are not regulated by genes but by the physical forces at play at that scale. 

**Figure 8:**
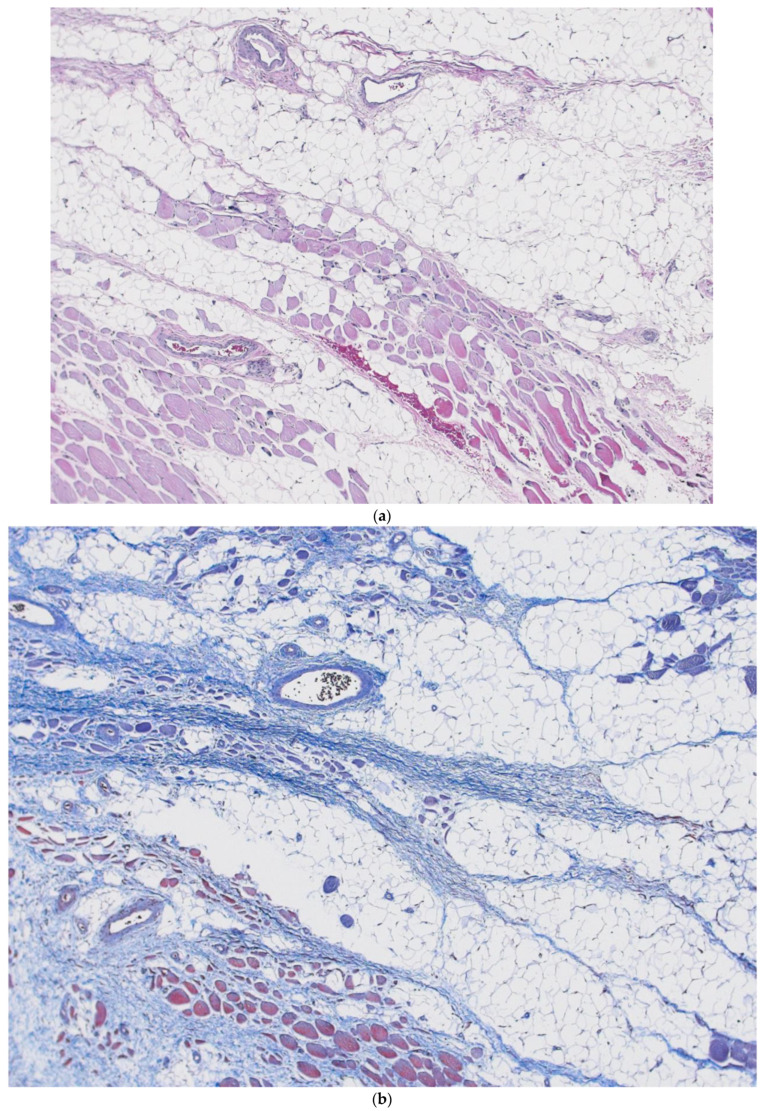
(**a**) Interface between superficial and deep fascia and muscle, illustrating the stacked layers, or lamina, with loose areolar tissue, or interstitium, between the layers. (**b**) Interface between superficial and deep fascia and muscle, illustrating the stacked layers, or lamina, with loose areolar tissue, or interstitium, between the layers. Magnification 100×.

Hyaluronan (HA) is integral to morphogenesis. Spicer and Tien state that HA is one of the most functionally versatile biological polymers [26]. It can have multiple functions depending on its large polymer-synthesized form or smaller forms attained by various collagenase enzyme degradations. Because of the large amount of space that the polyanion occupies, HA can not only attach to cells tethering matrices located on the cell membrane, but it can attach to multiple adjacent cells, connecting them and holding them in place. Smaller, low molecular weight versions of HA can act differently as they have signaling properties. Hyaluronan came onto the scene late in the metazoan evolution and appears to be important to vertebrates’ emergence [27]. It is known to be important in chondrogenesis and joint development, where condensation of mesenchymal cells that differentiate into chondrocytes then activate the HAS2 gene, which codes for hyaluronan synthase 2, which is responsible for HA production in the cell membrane and its transport into the ECM. Local production of HA allows chondrocytes to separate by release of the cell–cell contacts. These chondrocytes then enter a high-HA-production phase, which separates the groups of cells into layers in a process called cavitation, thus forming a cell-free space and layers that will eventually become the joint [26]. One cannot help but wonder if such a separating process like this, also described similarly by Blechschmidt, might not be important to the self-organizing basic fascia building block. Could fibroblast, or fibroblast-like cells, similarly shift into a high production mode of hyaluronan production after they had condensed into two-dimensional sheets, separating these fascia sheets into stacked layers? If this is true, and such sequences are important to regenerative healing, then reservoirs of interstitium would have to be ubiquitous and near any area of potential injury (Figure 8a,b). The interstitium would be perfectly situated to facilitate immune cell trafficking, such as monocyte and macrophage migration, to the regeneration site. 

A beautiful narrative of injury/regeneration can be described: Trauma or surgery divides tissues and opens blood vessels. Extravasation of blood outside the arterial lumen floods into the accompanying interstitium, which dynamically expands to accommodate the flow. Platelets in the blood encounter tissue-factor-presenting cells and thrombin, which activate them into releasing their factors, leading to platelet/fibrin clots. The clots and vasoconstriction stop the bleeding, but not before the interstitium has been flooded, causing inflammation, swelling, and ecchymosis. (Theise points out one key function of the interstitial tissue is to accommodate dynamic changes in the volume of extracellular fluid. Perhaps, this is why he has observed no continuous lining of the space with an endothelium, which could only swell so much before exceeding its limits and bursting.) Inflammatory-mediated diapedesis brings immune cells and more edema fluid into the injured tissue spaces, creating an opportunity for regeneration. The first few days to one week are characterized by this proinflammatory (M1-like) phase, in which macrophages clear the debris, and immune cells attack any infectious agents. By the start of the second week, under ideal circumstances, inflammation has abated, and swelling subsides, leaving a loose, low-tissue tension environment whose mechanotransduction effects cause macrophages to enter a pro-remodeling (M2-like) regeneration phase, demonstrating the dynamic interaction between the extracellular-matrix proteins and macrophage phenotypes [28]. The regenerative tissue milieu is characterized by an influx of macrophages from the surrounding superficial fascia remnants of the wound [29] and capillary ingrowth along the self-organizing collagen fascial scaffold (2-D sheets/vertical retinaculum). Su et al. showed the superficial fascia to be the origin of adipose cells in rat growth and development. They identified adipose precursor cells, resident in the fascial vasculature (wrapped with interstitium), as the source of adipogenesis. Male rats at one week old were studied by looking at the superficial fascia localized above the hindlimb using a stereomicroscope. They identified a region of fascia, without adipose tissue, characterized by blood vessels, collagen fibers, elastin fibers, fibrocytes, mast cells, and ground substance (i.e., the Interstitium). At seven weeks, they noted the formation of adipose lobules originating from the adventitia of the main blood vessel of the region and stated: 

“Adipose precursor cells reside in the vasculature of fascia and are physiologically active. During postnatal growth, adipose cells gradually arise from fascial preadipocytes to form primitive adipose lobules. At the lobule front, capillaries wrap around and pass ahead of adipocytes to form the distal neo vasculature, which might replenish the pool of preadipocytes and supply nutrients and hormones to ensure continuous adipogenesis in situ”. They also explained that as the two-dimensional sheets of adipocytes expand, some adipocytes overlay on top of other adipocytes, and a primitive three-dimensional tissue is formed in the fascia (presumably as the leading edge of neovascularization breaks into the *z*-plane along vertical retinaculum). They concluded the following: “Thereafter, this fascia naturally becomes the connective tissue continuum of renascent adipose tissue, and still preserves fascial preadipocytes and niche to ensure regeneration of adipocytes inside adipose tissue. With continuous adipogenesis, primitive adipose lobules in the superficial fascia, a subcutaneous nonadipose tissue, may gradually expand to form a rudiment of subcutaneous adipose tissue” [30].

**Figure 9:**
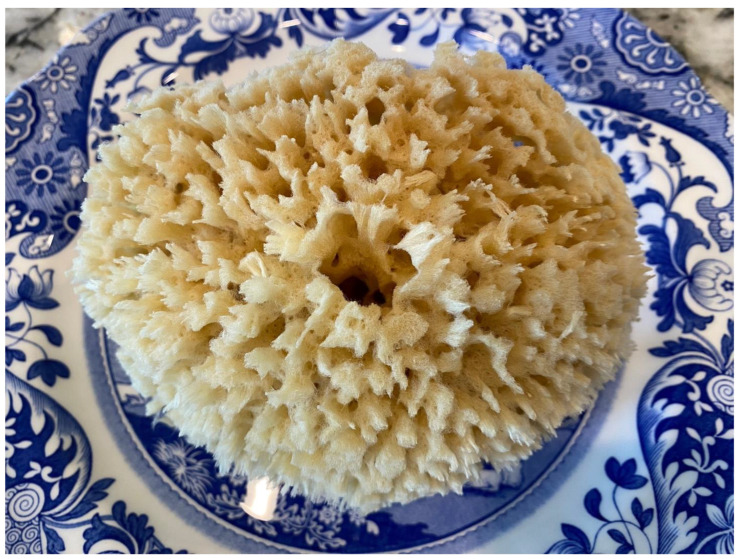
Natural sea sponge illustrating fractal branching “negative space” between the collagen scaffold.

The interstitium is an important part of the entire fascia system throughout the body. It is essentially an acellular space filled with proteins, GAG, and serum that is in the negative spaces of the body, or what is known in design as *white space*. Take, for instance, the purple and orange “Fed Ex” logo everyone is familiar with. The orange E and X outline a white arrow between the letters, pointing from left to right. This powerful impact on the design lets you know that FedEx is about moving things forward in business logistics. Similarly, the absence of cells in the *white space* of the interstitial system has a powerful impact on the body’s tissues. This negative space is a design solution used by the oldest known animals—sponges. Sponges have existed for over 500 million years and are too primitive to have specialized tissues or organ systems. However, they have a handful of specialized cells that generate and live on a scaffold made of a collagen-based protein called Spongin [31]. Most of us are familiar with natural bath sea sponges, which have been decellularized, leaving behind their collagen scaffold, used by the animal in place of a skeletal system, with the negative space inside the perforated body of the sponge in place of a cardiovascular system. This combination of collagen supports fibers and fractal, reticular open spaces make a bath sponge hold water and let it go when squeezed (Figure 9). 

The collagen of the human interstitium’s acellular negative space, which contains plasma and glycoproteins, instead of the seawater in sponges, can dynamically hold large volumes of fluid and allow the transport of cells and cytokines. It appears to be a prototype circulation system, or anlage, that more-advanced animal tissues have built on top of. This would explain the interstitium’s presence in the basic fascia segment throughout the body and the blood vessels and nerves it surrounds. 

Self-organization in complex systems is seen throughout nature, both inorganic and organic. The superficial fascia system appears to be no exception. A reactive media is required to set up the proper beginning environment, mixed with enough components to ensure complexity, followed by simple rules that guide the development of useful patterns. According to Adrian Bejan’s Constructal Law, “For a finite-size flow system to persist in time (to live), it must evolve such that it provides greater and greater access to the currents that flow through it” [14]. As an engineer, Bejan saw the same design patterns throughout nature but in different contexts as evidence that “the generation of images of design (pattern, rhythm) in nature is a phenomenon of physics”. Whether the patterns are of lightning strikes, river deltas, tree branching, artery/capillary branching, or collagen cross-linking, the result emerges and is a highly evolved solution to a flow-design problem solved by nature. Bejan, like D’Arcy Thompson a century earlier, reminds us that the evolution of biological form relies on genetics and physics. Once analogous patterns in nature, such as spongin scaffolds in ancient fossils, surface patterns of Illinois pondweed in lakes, bone trabeculae (spongy bone) with the reticular spaces of marrow, or the reticulated spaces in the human fascial interstitium are found, we can deduce that emergent self-organization is at play.

## 4. Clinical Implications

Various specialists in the manual healing arts have long known about the clinical implications of the fascial system in health and disease, as well as illnesses such as myofascial-pain syndrome. Fibromyalgia carries with it systemic symptoms, like viral prodromes. Interstitial cystitis and interstitial lung disease/pulmonary fibrosis involve inflammation, heightened sensory nerve response, and fibrosis, possibly mediated by abnormal myofibroblast of the interstitial layer. All these diseases are poorly understood or idiopathic and difficult to diagnose and treat. Improvements should be made with a better understanding of the fascial anatomy and its underlying interstitial system. 

As a surgeon specializing in plastic surgery, I can attest to the surgical implications of the fascia system from firsthand experience. Third-space fluid accumulation can be a result of systemic physiologic alterations known as anasarca, but more commonly it is the result of fluid resuscitation necessary because of hypovolemic shock caused by sepsis, trauma, or surgical blood loss. Since the mid-century, physiologic surgical research pioneered by Alfred Blalock on hypotension caused by blood loss, treatments have focused on intravascular volume replacement [32]. In severe blood loss, capillaries become leaky, and the fluid transfused to maintain adequate intravascular volume and blood pressure leaks into the interstitium. Fortunately, this special part of the fascia system can expand dramatically to accommodate these fluid shifts without cells bursting. This process, however, overwhelms the lymphatic system’s ability to reclaim the fluid lost from the intravascular space, leading to a recurrent loop of intravenous fluid infusion, leakage, and further hypovolemia. The backup leads to profound swelling of tissues throughout the body, which is evidence of the importance and ubiquity of the interstitium. Only once the shock has abated will the intravascular volume stabilize, along with the patient’s blood pressure, leading to a reversal of fluid movement from the interstitium and back into the intravascular space.

**Figure 10:**
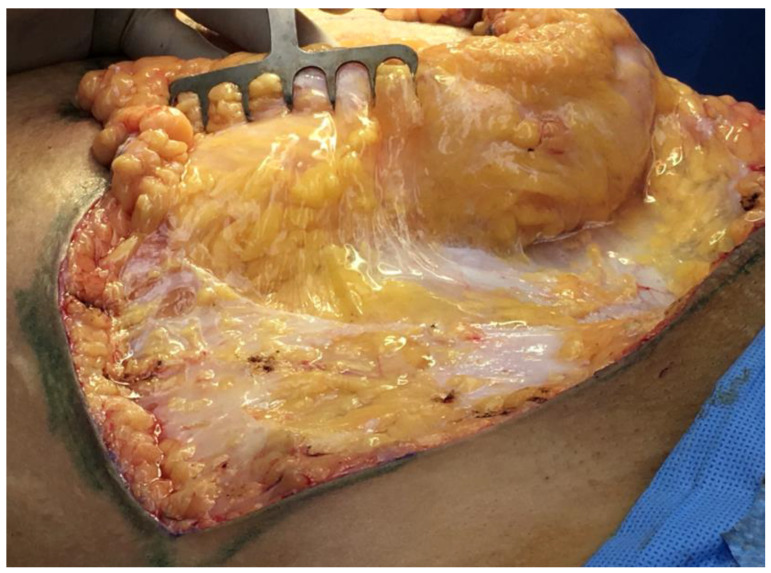
Abdominoplasty procedure with the pannus being resected and retracted upwards, away from the low abdominal incision in the lower right of the picture. Tumescent local anesthetic fills the interstitial space below the white Scarpa’s fascia, seen in the lower left of the picture. The clear, loose, areolar connective tissue keeps the tumescent fluid from running out of the wound.

As discussed earlier in this paper, the use of dilute local-anesthetic solutions with epinephrine, known as *tumescent anesthesia* (typically 1000 cc of saline solution, 30 cc of lidocaine 1% solution, and 1 milligram of epinephrine) is commonly used in plastic and reconstructive surgery. The volume injected through needles in facelifts (250 cc) is smaller than the large volumes (1–4 L) injected in liposuction, breast surgery, and abdominoplasty (Figure 10). 

The fluid spreads throughout the tissue target area by traveling through the horizontal layers of the interstitium, moving from deeper to subcutaneous locations by traveling through interstitial wrappings of the vertical vascular perforators, which climb along the vertical retinaculum. This is evidenced by the tumescent canula’s placement in the deeper fascial layers and the fluid’s rapid transport to all depths of the tissue. Vasoconstriction is witnessed as the epinephrine elicits a blanching of the subdermal plexus, usually within 5 min of injection. Just as in shock-related edema, it takes 24 to 48 h for the large volumes of fluid in the interstitium to reverse back into the bloodstream and edema to subside. Considering the lymphatics are backed up, the lidocaine must also persist in the target tissue, producing a long-lasting local pain block, which is ideal for outpatient plastic surgery procedures. 

The characteristic of the superficial fascia’s interstitium to act as a transportation zone for cells and other bioactive substances is a double-edged sword. It also means that metastatic cancer cells have an express lane to travel to regional sites within the body. Hyaluronan, which resides in the interstitium in high concentrations, is integral to the epidermal to mesenchymal transformation (EMT) [26], required for intravascular cancer cell metastasis and the direct spread through interstitial tissue transportation routes. However, similar activities are necessary for the regenerative healing of injured tissues. Perhaps, a better understanding of this underlying fascial anatomy will lead to the discovery of new cancer treatments!

## 5. Regenerative Medicine

Regenerative medicine and tissue engineering generate the stroma and parenchyma of tissues that may have been injured or lost because of disease, injury, or surgery. Regenerative tissue therapies are at the heart of long-hoped-for cures in medicine. The superficial fascia system, located beneath the skin with extensions into the underlying deep fascia, musculoskeletal, and visceral compartments, could act as a cellular *ecotone* for biological therapies. 

The superficial fascia system (SFS) is the basic model of connective tissue in the human body. It interconnects the overlying skin via the vertical retinaculum to the underlying muscle–skeletal system via zones of adhesion to the deep fascia, present since embryonic development. Connective tissue fascia continues beyond the superficial and muscular deep fascia into the organs, which it surrounds and defines with each organ’s stroma and capsules. Cells, supported by stroma, that act as metabolic factories are referred to as *parenchyma*. Cellular therapies seek to replace or augment parenchymal function, but they require stroma to act as a nest for these cells and provide a sufficient blood supply. Most cellular therapies require a volume of tissue that is too large to be implanted as free grafts; they require the generation of a blood supply, making the interstitium of fascia an ideal target for transplantation, as demonstrated in a mastectomy breast-reconstruction case, shown on YouTube (https://youtu.be/dHcRw35WAh0?feature=shared accessed on 21 November 2023). 

## 6. Anti-Aging

The superficial fascia has been the anatomic zone treated in cosmetic medicine. Primitive attempts at anti-aging therapies, using HA injectables and fat grafting have already proved wildly popular with patients and doctors alike. Humans age in predictable ways that have typical stigmata. In the face, there is an involution of the soft tissues of the temples, cheeks, and neck, beginning in the 50 s and 60 s. This has been explained by preadipocyte supplies in the superficial fascia wearing out, leading to the involution of facial fat, deflation, and sagging. Plastic surgeons have historically blamed sagging on weakening dermis, but this cannot be the only reason. Hyaluronan plays in so many functions of the fascia; HA is a critical component of plump and taught skin with youthful elastic recoil, which can rebound into repose after expressive movement. It is also important to youthful skin’s ability to resist gravity. As our understanding of this important anatomy expands, with a better knowledge of its collagen and glycosaminoglycan constituents, we will no doubt continue to improve surgical and non-surgical aging treatments. 

## 7. Conclusions

The fascial interstitium is like the Amazon River, which flows through a dense jungle of parenchymal cells, provides for cell trafficking, and aids the currents of metabolites in their flow from capillary to cell and on to lymphatic uptake. Like a river, it swells and overflows the banks when inflammation leads to leaky capillaries. And, just like the flow of the Nile which sends nutrient-rich silt into its river basin, the interstitium is the site of the accumulation of proteins like fibronectin, platelets, immune cells, chemokines, and cytokines, and the trafficking of macrophages, which provide the raw material for regenerative new growth, stimulated by capillary branching which creeps into these protein-rich spaces, along a 3D grid of self-organizing collagen. The interstitium can enlarge like a flood plain in the spring melt from upstream headwaters. Room to grow and the requisite blood supply provided by branching capillaries, facilitated by hyaluronan, are all provided by the interstitial regions of fascia—this most amazing autopoietic tissue system. 

## Data Availability

Please contact the author at robertrehnke@me.com to receive copies of live surgical video data from our operating room which in detail support the opinions of the author put forth in this commentary article.

## Supplementary Materials

The following supporting information can be downloaded at https://www.mdpi.com/article/10.3390/life14010089/s1, Video S1: Basic Body Segment-Abdomen; Video S2: Superficial Fascia of the Temple; Video S3: Vertical Mastopexy.
